# Abstract of the Proceedings of the Racine Medical Association

**Published:** 1863-03

**Authors:** John G. Meachem

**Affiliations:** Secretary; Racine


ART. III.—*Abstract of the proceedings of the Racine Medical Association.*

TUESDAY EVENING, February 10th 1863.

Dr. John L. Page, in the chair. Drs. Nettleton, Smith, Thompson, Wadsworth, Hoy, Peak, and Meachem, were also present.

*Dr. Hoy* read a very able paper on vaccination, in which he gave much valuable statistical information. It was his opinion that re-vaccination should be performed, at least, once in five years. Sufficient care was not taken in the selection of pure vaccine virus, and the general prevalence of variola throughout the United States, for the past few years, was attributable to this cause. Many families called a physician, and had one member vaccinated, and to save expense, all the other members were vaccinated from this one, by some person unused to the business, and who could not tell a spurious from a genuine vesicle. They all fancied themselves secure from variola, in any, and all forms, when in fact, perhaps not one of them had had the true vaccine disease.

He believed it necessary in preparing the scab for future use, to scrape off its under surface, as it consisted of nothing but pus, and was consequently worse than useless. The scab should also be well dried before being encased in wax, as it was less likely to become inert by long keeping.

*Dr. Wadsworth* thought it quite unnecessary to re-vaccinate so often. He was quite inclined to the opinion that if the genuine vaccine disease had once been produced in a system, it was for ever a protection; at least as much so as an attack of variola could be to a subsequent attack. He had been vaccinated but once, which was in infancy, and, though he had been exposed to variola many times during the course of his long practice, he had never taken the disease.

*Dr. Smith* thought that if a second vaccination took effect, it was pretty good proof that the first would have been no protection.

*Dr. Thompson* had seen two attacks of variola in the same individual, with several years intervening.

Diphtheria, the subject appointed for discussion, at this meeting, was then taken up.

*Dr. Wadsworth* said that diphtheria was represented by some medical men, and by quacks generally, to be greatly prevalent at this time. Every case of sore throat was so called, creating a panic in community, though in these cases there was no exudation, or other signs of the true disease. This was an attempt to build up a reputation upon a false, and criminal basis, and should be frowned upon by medical men. He had watched every case of scarlet fever, of late, to see if he could discover the diphtheritic exudation in the anginose affections of that disease, but he had not observed it in a single instance. It might possibly take on this character when diphtheria was prevailing as an epidemic, but otherwise thought it rarely observed

*Dr. Page* believed diphtheria to be the same disease which has heretofore prevailed as an epidemic, known to the profession by the names of angina, and cynanche maligna, and popularly as "malignant sore throat." The terms angina, from the Latin, signifying to choke; cynanche, from the Greek, to suffocate, and diphtheria, a membrane, convey no correct idea of the nature of the disease, but rather represent symptoms which sometimes attend other diseases—scarlet fever and croup. Diphtheria is not a local, but a constitutional disease, and is, in his opinion, infectious. Local applications, either externally, or to the fauces, by means of the probang or camels hair brush, except in the first stage, usually do but little good, and may do much harm. He here stated a case, where, upon the use of the sponge probang, saturated with a preparation of iron, a portion of the leathery exudation fell into the glottis, and the little patient suffocated.

It is usual in domestic practice, to make a variety of applications to the external throat, much to the discomfort of the patient. He removed all these, and, if he saw the case early, before the exudation, or when it was just commencing, he applied, by means of the pencil, a strong solution of nitrate of silver. In most cases, and especially when much fœtor existed, he prescribed either chlorate of potash, or chlorinated soda in solution, for a gargle; the former also to be taken internally. He relied mainly upon chlorate of potash, ses qui chlo, iron, quinine, iodid potassium and nutrition, *pro re nata.* Some cases yielded readily to simpler, almost *negative* treatment, others required a sustaining positive treatment, *ad initio.*

Scarlatina and diphtheria frequently co-exist, or rather the former puts on the type of the latter. He was then treating a case of scarlatina, in which the exudation was abundant. He cited many cases illustrative of his views.

*Dr. Meachem* regarded diphtheria as contagious, though not as much so as scarlatina, measles or variola. He was then treating a young lady, 16 years of age, who had been residing several miles from the city, where not a case of diphtheria had occurred. She came to town to assist in nursing a young nephew, who was suffering from a severe attack. She contracted it in a week, aud passed through a very alarming course of the disease, furnishing throughout an extraordinary amount of the diphtheric exudation.

In relation to treatment he would say a few words. Many cases he had, no doubt, proved fatal, from not sufficiently sustaining them. It is many times difficult to get little patients to take half the nourishment that is really required. This is a point that should be daily inquired into by the attending physician. His plan of treatment was to give an emetic of ipicac, as early as possible, brush the tonsils, palate and fauces, with Squibbs' sol. of persulphate of iron, a suggestion he received from Professor J. P. White, of Buffalo, about two years ago. Give internally the following mixture:

Chlorate Potass, ʒ j

Aqua Pura, ℥ ij

Tr. Ferri Mur, f ʒ j

Acid Mur, gtts v

Misce.

A teaspoonful to be given once in four hours; and after the disease has advanced a day or two, a powder of quinine between each dose. Milk punch, strong beaf tea, &c., was not only allowed, but urged upon his patients,

He had used the nitrate of silver as a local application, but regarded the sol. of persulphate of iron as decidedly to be preferred. He had also used Creosote, and liked it very much, when there was much fœtor present. The effect of the emetic in the early stages was very beneficial. It equalized the circulation, and unlocked the secretions generally—important results to be obtained.

He would say that he now never used the sponge probang for making the local applications, but always the camel's hair brush.

*Dr. Peak* had been in the habit of using as a local remedy, Chlo. Potass, Sub. Borate Soda, and Sugar of Milk, of each, equal parts, well triturated together, a few grains of which was allowed to dissolve upon the tongue several times a day.

*Dr. Nettleton* had treated an epidemic many years ago, in Miss., what he and other physicians then called Cynanche Maligna, but which he now knew to have been diphtheria.

He read a very able article on the disease, which should be published entire.

JOHN G. MEACHEM, M. D., Secretary.

*Racine*, February 28, 1863.

